# Differentiating Peripherally-Located Small Cell Lung Cancer From Non-small Cell Lung Cancer Using a CT Radiomic Approach

**DOI:** 10.3389/fonc.2020.00593

**Published:** 2020-04-22

**Authors:** Bihong T. Chen, Zikuan Chen, Ningrong Ye, Isa Mambetsariev, Jeremy Fricke, Ebenezer Daniel, George Wang, Chi Wah Wong, Russell C. Rockne, Rivka R. Colen, Mohd W. Nasser, Surinder K. Batra, Andrei I. Holodny, Sagus Sampath, Ravi Salgia

**Affiliations:** ^1^Department of Diagnostic Radiology, City of Hope National Medical Center, Duarte, CA, United States; ^2^Department of Medical Oncology and Therapeutics Research, City of Hope Comprehensive Cancer Center and Beckman Research Institute, Duarte, CA, United States; ^3^Applied AI and Data Science, City of Hope National Medical Center, Duarte, CA, United States; ^4^Division of Mathematical Oncology, City of Hope National Medical Center, Duarte, CA, United States; ^5^Hillman Cancer Center, University of Pittsburgh Medical Center, Pittsburgh, PA, United States; ^6^Department of Radiology, University of Pittsburgh Medical Center, Pittsburgh, PA, United States; ^7^Department of Biochemistry and Molecular Biology, University of Nebraska Medical Center, Omaha, NE, United States; ^8^Department of Radiology, Memorial Sloan-Kettering Cancer Center, New York, NY, United States; ^9^Department of Radiation Oncology, City of Hope National Medical Center, Duarte, CA, United States

**Keywords:** small cell lung cancer (SCLC), non-small cell lung cancer (NSCLC), computed tomography radiomics (CT Radiomics), non-linear classifier, artificial neural network

## Abstract

Lung cancer can be classified into two main categories: small cell lung cancer (SCLC) and non-small cell lung cancer (NSCLC), which are different in treatment strategy and survival probability. The lung CT images of SCLC and NSCLC are similar such that their subtle differences are hardly visually discernible by the human eye through conventional imaging evaluation. We hypothesize that SCLC/NSCLC differentiation could be achieved via computerized image feature analysis and classification in feature space, as termed a radiomic model. The purpose of this study was to use CT radiomics to differentiate SCLC from NSCLC adenocarcinoma. Patients with primary lung cancer, either SCLC or NSCLC adenocarcinoma, were retrospectively identified. The post-diagnosis pre-treatment lung CT images were used to segment the lung cancers. Radiomic features were extracted from histogram-based statistics, textural analysis of tumor images and their wavelet transforms. A minimal-redundancy-maximal-relevance method was used for feature selection. The predictive model was constructed with a multilayer artificial neural network. The performance of the SCLC/NSCLC adenocarcinoma classifier was evaluated by the area under the receiver operating characteristic curve (AUC). Our study cohort consisted of 69 primary lung cancer patients with SCLC (*n* = 35; age mean ± SD = 66.91± 9.75 years), and NSCLC adenocarcinoma (*n* = 34; age mean ± SD = 58.55 ± 11.94 years). The SCLC group had more male patients and smokers than the NSCLC group (*P* < 0.05). Our SCLC/NSCLC classifier achieved an overall performance of AUC of 0.93 (95% confidence interval = [0.85, 0.97]), sensitivity = 0.85, and specificity = 0.85). Adding clinical data such as smoking history could improve the performance slightly. The top ranking radiomic features were mostly textural features. Our results showed that CT radiomics could quantitatively represent tumor heterogeneity and therefore could be used to differentiate primary lung cancer subtypes with satisfying results. CT image processing with the wavelet transformation technique enhanced the radiomic features for SCLC/NSCLC classification. Our pilot study should motivate further investigation of radiomics as a non-invasive approach for early diagnosis and treatment of lung cancer.

## Introduction

Lung cancer is the second most commonly diagnosed cancer for both men and women, representing around 13–14% of yearly cancer diagnoses for both genders. It is also the leading cause of cancer mortality, accounting for about a quarter of all cancer-related deaths worldwide (1). There are two major types of lung cancer: small cell lung cancer (SCLC)—the aggressive lethal neuroendocrine carcinoma that accounts for ~10–15% of all lung cancer cases—and non-small cell lung cancer (NSCLC), which accounts for 85% of all lung cancers (2). As a class, NSCLC broadly includes adenocarcinoma, squamous cell carcinoma, and large cell carcinoma (3). NSCLC can be divided into subclasses based on the presence of driver mutations in proteins such as epidermal growth factor receptor (*EGFR*), anaplastic lymphoma kinase (*ALK*), and Kirsten rat sarcoma virus (*KRAS*). Treatment options and survival largely depend on the type of lung cancer (4–6). The new standard of care for advanced SCLC consists of a combination of carboplatin, etoposide and immunotherapy; however, chemoradiation, targeted therapies, and immunotherapy are the treatment options available to patients with advanced NSCLC (5, 7). For patients with locally advanced disease or distant metastases, the 1-year survival rate is 15–19% for NSCLC and <5% for SCLC (8, 9). In the context of personalized medicine for NSCLC, targeted therapies for common driver mutations, and immunotherapy targeting the PD-1 receptor and its ligand PD-L1 have shown promising data for improving treatment and survival (10, 11). However, the primary factor in survival for both SCLC and NSCLC is early diagnosis that can be facilitated by an identification of radiologic phenotypes for the primary lung cancer subtypes.

Lung CT scan is the most commonly used imaging tool for lung cancer diagnosis. Multiple lung CT imaging characteristics that may help predict cancer have been identified in lung nodules. The commonly used imaging characteristics include the following: large nodule size; change in the size of the nodules over time; number and density of the nodules; and morphological signs of aggressiveness including irregular shapes and spiculated margins of the nodules (12, 13). However, CT imaging features for lung cancer are limited in number and the results from traditional CT imaging analysis are subjective because it relies on visual inspection by imaging specialists, potentially causing inter-observer variability (14). In addition, traditional CT analysis is limited in its ability to differentiate SCLC and NSCLC because of overlapping CT features. Both SCLC and NSCLC could present with spiculation, and could be associated with ground glass opacity or pleural reaction, which makes visual differentiation challenging in clinical practice. Biopsy is used to supplement CT imaging and to confirm the diagnosis when lung cancer is suspected. However, both bronchial brushing and CT-guided biopsy are associated with risks such as post-procedure infection, bleeding, and pneumothorax. In addition, pathological diagnosis through invasive biopsy is usually obtained from a focal area or areas of the tumor rather than the entire tumor, thus lacking the overall tumor characterization. Besides, biopsy results are not always promptly available. Therefore, it is prudent to develop non-invasive complementary approaches such as radiomic methods to differentiate primary lung cancer subtypes.

Radiomics is a computerized quantitative image analytical method that extracts large number of features from radiographic medical images using computing algorithms (15, 16). It converts an image database into a set of quantitative radiomic features that characterize the tumor heterogeneity regarding textural pattern, morphology in shape and geometry, and intensity in histogram-based statistics (17). Radiomic analysis of medical images generates reproducible quantitative image features, which could capture tissue microstructural patterns associated with genetic and proteomic signatures contributing to the biological basis of the disease (15, 18, 19). Aerts et al. identified an association between intratumoral heterogeneity reflected by radiomic features and the underlying gene expression patterns in their radiogenomic study of patients with lung cancer and head-and-neck cancer (17). Other researchers have shown that textural features depicting spatial heterogeneity in tumors could reflect genomic and phenotypic tumoral characteristics (19, 20). Radiomics has also been used to classify various NSCLC subtypes and SCLC based on lung CT images (21, 22). These promising initial results have motivated further research to develop non-invasive imaging methods to differentiate primary lung cancer subtypes for the purpose of early diagnosis and targeted therapy. There is extensive literature on radiomic research of NSCLC. For example, recent studies have shown that the NSCLC histologic subtypes could be effectively classified using a CT radiomic method (23–25). In addition, PET-CT radiomics could be used to differentiate between primary NSCLC and its metastasis (26). However, there is limited research focusing on differentiating SCLC from NSCLC, which is clinically relevant as early diagnosis and treatment of the two primary lung cancer subtypes can significantly improve prognosis.

Here, we used a radiomic approach to evaluate tumor heterogeneity of SCLC and NSCLC adenocarcinoma. We hypothesized that CT radiomics would provide distinctive features reflecting tumor heterogeneity for predictive classification of SCLC vs. NSCLC adenocarcinoma. We aimed to identify quantitative radiomic features for further evaluation as non-invasive imaging biomarkers. Such biomarkers could potentially be used to predict the pathological subtypes of primary lung cancer and to provide valuable information for early diagnosis and treatment of lung cancer.

## Materials and Methods

### Participants

We retrospectively identified patients with pathology-confirmed primary lung cancer who were treated at City of Hope (Duarte, CA) from 2009 to 2017. We identified patients with SCLC first and then matched these to patients with NSCLC during the same study interval. Post-diagnosis pre-treatment lung CT images were used for this study.

To be eligible for this study, patients with pathology-confirmed SCLC or NSCLC adenocarcinoma needed to have at least one pre-treatment lung CT scan showing a peripherally-located lung cancer. The peripherally located lung cancers in our study were defined to be the lung cancers located in the periphery of lung and being separate from the central structures such as the mediastinum and hilar structures. We selected peripherally-located lung cancers because of the clear tumor delineation from adjacent low-density lung parenchyma on lung CT images. We did not select centrally-located lung tumors because of the difficulty in identifying tumor boundaries from the adjacent mediastinum or hilar vasculature and lymph nodes due to similarities in tissue densities on the CT images without intravenous administration of contrast. The exclusion criteria included: treatment such as chemoradiation or surgery started before the lung CT scan, suboptimal lung CT quality due to respiration or other imaging artifacts, or having only centrally-located lung cancers. This study was approved by the Institutional Review Board at City of Hope National Medical Center. Informed consent was waived due to the retrospective nature of this study.

### Lung Tumor Segmentation

We retrieved the patients' lung CT images from the City of Hope Picture Archiving and Communication System (PACS) database, which were archived in three-dimensional (3D) volumes in a matrix size of 512 × 512 × 355 with a voxel size of 0.76 × 0.76 × 1 mm^3^. The lung CT scan was obtained in a GE CT 750HD with a scanning protocol including the following: 120 kV, 150–600 Auto mA (Tube Modulation), 0.5 s tube rotation, 40.0 mm coverage, helical scan (1.375:1/55 Pitch/Speed), coverage speed 110.00 mm/s and field of view with skin-to-skin coverage.

The lung cancers from the CT lung window images were initially segmented semi-automatically using the ITK-SNAP software (http://www.itksnap.org/pmwiki/pmwiki.php) by the trained research staff (NY, ZC, and BC). The supervising study radiologist (BC) is a board-certified radiologist with over 10 years of experience working on lung cancer imaging. This semi-automatic approach identified the locations of the tumors by indicating the region of interest (ROI) on the lung-window CT images and this approach should help to reduce the potential inter-observer or intra-observer bias. Subsequently, the tumors were then carefully assessed and delineated slice-by-slice by the trained postdoctoral fellow (NY) who is a physician with imaging training and who has traced tumors for radiomic research for 2 years, and by the staff scientist (ZC) who has had over 15 years of experience in imaging research. The study radiologist (BC) and the research team had joined sessions to visually re-check slice-by-slice of all tumor segmentations in a magnified display for reduction of delineation errors and for trouble shooting potential issues during tumor segmentation.

To evaluate the reproducibility of inter-observer and intra-observer tumor segmentation, we randomly selected 25 patients consisting of 13 SCLC patients and 12 NSCLC patients from our study cohort. Two trained researchers (NY and ZC) segmented the tumors independently and the two researchers were blinded to each other's segmentations for assessing the inter-observer consistency. In addition, one of the researchers (NY) repeated the tumor segmentation 1 week later to assess the intra-observer consistency. Both the inter- and intra-observer agreement for tumor segmentation was assessed by inter- and intra-class correlation coefficients (ICC). An inter-observer or intra-observer ICC >0.80 indicated a good agreement for tumor segmentation.

The inter-observer ICC between the two researchers (NY and ZC) for tumor segmentation achieved 0.97 ± 0.05 ranging from 0.93 to 0.99. The intra-observer ICC between the two measurements by the same researcher (NY) was 0.98 ± 0.03 ranging from 0.96 to 1.00.

The results indicated favorable inter- and intra-observer reproducibility and stability for tumor segmentation and subsequent radiomic feature extraction.

**Figure 1:**
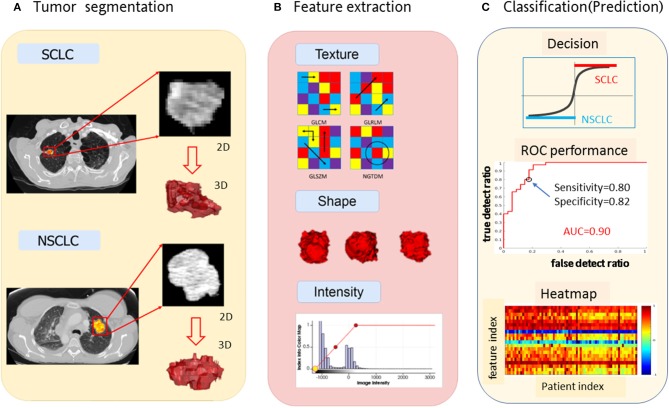
Schema for lung cancer segmentation, radiomic feature extraction and predictive modeling. **(A)** Representative CT images from small cell lung cancer (SCLC) and non-small cell lung cancer (NSCLC) showing tumor segmentation. **(B)** Illustrations of radiomic feature extraction for texture, shape, and intensity. **(C)** Decision of SCCL/NSCLC classification (upper panel) with the receiver operating characteristic (ROC) curves (middle panel) and the heat map of radiomic features (lower panel).

In Figure 1, we presented the overall schema for data analysis. Figure 1A presents the lung tumor segmentation. Next, radiomic features were extracted via tumor image analysis for texture, shape, intensity (Figure 1B). Finally, the SCLC/NSCLC classification was performed and statistically assessed in the receiver operating characteristic (ROC) curve (Figure 1C).

### Radiomic Feature Extraction

#### Histogram-Based Global Features

An image intensity histogram was generated for each 3D tumor image. We derived 8 statistical quantities from each histogram: max, min, range (max-min), mean, entropy, variance, skewness and kurtosis. Since there was no spatial information in the histograms, the histogram-inferred values were considered global features. During tumor image analysis, we retained the image intensity in original CT number, which informed on the tumor tissue radiodensity in reference to water at 0 (in Hounsfield unit). A high CT number in a tumor image may indicated fibrosis or calcification within the tumors.

#### Textural Features

Textural features may represent tumor heterogeneity. We extracted the tumor textural features using the MATLAB radiomic package (https://github.com/mvallieres/radiomics) and the textural analysis formula (27). Given a 3D tumor image, we first generated the textural matrices: gray-level co-occurrence matrix (GLCM), gray-level run-length matrix (GLRLM), gray-level size-zone matrix (GLSZM), and neighborhood gray-tone difference matrix (NGTDM). We then derived various textural features from these textural matrices. Specifically, we calculated 9 gray-level co-occurrence features from the GLCM matrix, 13 run-length features from the GLRLM matrix, 13 gray-level size zone features from the GLSZM matrix, and 5 neighborhood gray-tone difference features from the NGTDM matrix. We therefore obtained 40 textural features (= 9+13+13+5) from one tumor image.

During tumor image preprocessing, we re-sampled the image intensity with multiple quantization levels (denoted by Ng, a bin number of intensity range). For example, with Ng= {16, 32, 64, 96}, we repeated the textural feature extraction procedure 4 times and obtained a total of 196 image features (= 48 × 4, comprising 8 global features and 40 textural features). The Ng variable was used to find the optimal image digitization with reduced gray levels with the Lloyd-max' algorithm adaptive quantization method (28). Multiple Ng values yielded a large number of image features, which had considerable redundancy. Of these features, we selected a few important high discriminative features through a feature selection procedure, thereby empirically optimizing the Ng settings.

#### Wavelet Transformation

We first applied 3D wavelet transformation to each 3D tumor image to decompose it into 8 subbands (29), denoted by {LLL, LLH, LHL, LHH, HLL, HLH, HHL, HHH}, where L and H denoted low-pass and high-pass filtering along one dimension. Then, we conducted inverse 3D wavelet transforms for individual subband image reconstruction using the same wavelet kernel. For each reconstructed subband image, we repeated the procedures for extracting histogram-based global features and textural features. As such, the number of features was multiplied by 8-fold corresponding to 8 wavelet subbands.

### Feature Selection

Feature selection and measurements in this study were performed with respect to a specific parameter. For example, the intensity range max-min constituted a vector, called a feature vector. Each feature vector was normalized by max = 1 (feature vector divided by its maximum entry). There existed considerable redundancy among the feature vectors. To correct the issue of redundancy and to create a two-class (SCLC/NSCLC) classifier, we estimated the feature classification performance (also known as feature relevance) by evaluating the correlation between the feature vector (a sequence of feature values across the cohort) and the classification target vector (composed of entries representing the pre-defined target classes: SCLC = 1 and NSCLC = 0), denoted by corr (correlation in range [−1,1]). We used mutual information to analyze the redundancy and dependence among features.

During the feature selection procedure, we used a minimal-redundancy-maximal-relevance method (mRMR) to remove the redundant and less-relevant features (30). In implementation of mRMR, we iteratively deselected the features based on a redundancy minimization of the mutual information among features and a relevance maximization of the mutual information between the selected features and the pre-defined target classes, until the feature number reduced to 20 (empirically specified). After that, the top 20 radiomic features out of 1,731 features were then selected for building the SCLC/NSCLC classification model.

### Non-linear Classification With Artificial Neural Network

**Figure 2:**
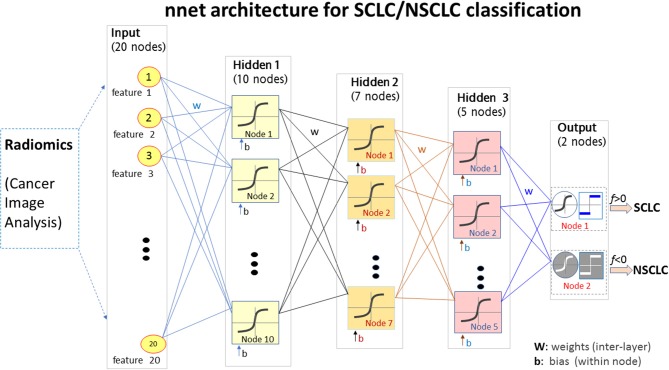
The *nnet* architecture of the radiomics-based SCLC/NSCLC classifier. This figure presents the input layer with 20 nodes receiving 20 radiomic features, the 3 hidden layers for non-linear mapping, and the output layer with 2 nodes for “SCLC” and “NSCLC” decision upon a hard thresholding f(node)>0 and f(node)≤0, respectively. SCLC, small cell lung cancer; NSCLC, non-small cell lung cancer.

Using the top 20 radiomic features, we constructed a multilayer neural network (*nnet)* using the MATLAB procedure *nnet* = *patternnet* (10, 7, 5), which consisted of 3 hidden layers with 10, 7, and 5 hidden neurons (nodes) in a sequential order (https://www.mathworks.com/help/stats/machine-learning-in-matlab.html). The *nnet* architecture was presented in Figure 2. The input layer consisted of 20 neurons receiving the 20 feature values, and the output layer consisted of 2 layers indicating separated SCLC class (in label 1 for the thresholding f(node)>0) and NSCLC class (in label−1 for the thresholding f(node) <0). The non-linear mapping from 20 input nodes to 2 output nodes involved diverse settings such as logistical mapping (2-class problem), nodal sigmoidal activation, internetwork weights, and biases which were integrated in the *nnet* configuration.

The *nnet* training process was performed with random initial weights and biases prior to iteration on feed forward, nodal non-linear activation, and error backpropagation (https://www.mathworks.com/help/stats/machine-learning-in-matlab.html). We specified the training function as “*trainlm*” with the multivariate Levenberg-Marguardt algorithm (29), and the activation function as “*tanh*” with a hyperbolic tangential sigmoid function, and a maximum iteration of 1000 epochs and a control error < 10^−3^.

With the *nnet* architecture and the radiomic feature set, we developed a primary lung cancer classifier for SCLC/NSCLC discrimination by rendering training, validating, and testing procedures repeatedly. During the training stage, the cohort dataset (*n* = 69) was randomly decomposed into three subgroups: training (49–70% total), validation (10–15% total), and testing (10–15% total). For example, we preset a sample split by an allocation ratio “training 70%, testing 15%, validation 15%.” During *nnet* training, the sample set was randomly partitioned by the preset allocation ratios: 70% training +15% validation +15% testing. The sample set partition could be specified with other allocation settings during the *nnet* configuration. The validation and testing procedures were carried out using 15% sample patients (~10 patients); this number of patients was randomly selected by data shuffling in multiple repetitions. Therefore, one patient was allocated to the “training” cohort at one run and the same patient could then be allocated to the “validation” cohort at next run or to the “testing” cohort at next run as the random allocation process continued. The validation subgroup was necessary to avoid potential overfitting during the *nnet* training. The classifier performance was further evaluated with the testing subgroup which was an independent group reserved for the testing purpose during random allocation of the cohort.

By fixing the random number generation (*rng* (“default”) in MATLAB), the *nnet* classifier was reproducible for each (training+validation+testing) trial. When the random initialization (for *nnet* weights and biases) was not fixed, the *nnet* classifier yielded variations from trial to trial. We repeated the (training+validation+testing) procedure 30 times and evaluated the classifier performance by averaging the results of the 30 trials.

In addition to the image features, we also collected the patients' clinical and demographic data including age, gender, smoking status and race (also denoted as clinical features). We included these clinical features into the classification of the SCLC/NSCLC discrimination depending on their classification performance.

The SCLC/NSCLC differentiation may be implemented using diverse pattern classification methods with radiomic features. For example, one may use a linear discrimination analysis and a support vector machine method to bipartite the high-dimensional features into SCLC and NSCLC categories. For the SCLC/NSCLC classification (a typical 2-class problem) from high-dimensional features in a number of tens to thousands as in our study, we used multilayer artificial neural network classifiers (https://www.mathworks.com/help/stats/machine-learning-in-matlab.html), which in principle could achieve more optimal arbitrary non-linear mapping (e.g., non-linearity beyond analytic description or mathematical tracking) with appropriate configuration and training.

### Statistical Analysis

The classification performance of the SCLC/NSCLC classifier was evaluated using the area under the receiver operating characteristic (ROC) curve (AUC) during the testing stage. From the ROC curve, we calculated the AUC values and identified the sensitivity/specificity at a point on the curve around 10:30 o'clock position to quantify the classification performance. In addition to performing ROC analysis on each (training+validation+testing) trial, we used the average of 30 trials (generated with random initializations for *nnet* training) to report the overall performance of the SCLC/NSCLC classifiers. The classifier performance was statistically assessed by the standard ROC method, which involved the statistical comparison between the *nnet* output classes and the pre-defined target classes.

Categorical variables such as gender, history of smoking and race between the SCLC group and the NSCLC group were tested using Chi-square tests. Two-sample *t*-tests were used to compare the group differences (SCLC/NSCLC) for a continuous variable such as age. *P* < 0.05 was considered statistically significant.

## Results

### Patient Information

Our study consisted of 69 primary lung cancer patients with SCLC (*n* = 35, age range [46, 81] years, mean± SD = 66.91 ± 9.75 years), and NSCLC adenocarcinoma (*n* = 34, age range [36, 85] years, mean ± SD = 58.55 ± 11.94 years). The SCLC group consisted of a higher percentage of male patients and smokers (*p* < 0.05). The patient demographic data are presented in Table 1. There were statistically significant differences between the SCLC group and the NSCLC group regarding age (p = 0.002) and race (p = 0.03), as determined by the default significance level at p < 0.05.

**Table 1:** Patient demographic data.

	**SCLC**	**NSCLC**	***p***
	***N* = 35**	***N* = 34**	
Gender			0.01
Male	24 (68.57%)	12 (35.29%)	
Female	11 (31.42%)	22 (64.70%)	
Age			0.002
Mean ± SD	66.91 ± 9.75	58.55 ± 11.94	
History of Smoking			<0.001
Yes	34 (97.14%)	9 (26.47%)	
No	1 (2.86%)	25 (73.53%)	
Race			0.03
Asian	7 (20.00%)	16 (47.05%)	
Caucasian	26 (74.29%)	15 (44.12%)	
Other	2 (5.71%)	3 (8.82%)	

*NSCLC, non-small cell lung cancer; SCLC, small cell lung cancer*.

### Feature Extraction

For feature extraction, we obtained a total of 48 features (8 histogram features, 40 textural features) from each original tumor image prior to preprocessing. After tumor image intensity re-quantization by Ng = {16, 32, 64, 96}, we obtained 192 (48 × 4) additional features. By incorporating a 3D wavelet transformation, we obtained 1728 (= 192 × 9) features. Including the clinical features (age, gender, and smoking status), we obtained a total of 1,731 features (=192 × 9+3). Supplementary Figure 1 presents a heat map of all radiomic and clinical features. Supplementary Figure 2 contains the mutual information map for the features in a 1731 × 1731 symmetric matrix, as shown in the upper triangle. A large mutual information value indicated a high redundancy between the features.

### Feature Selection

**Figure 3:**
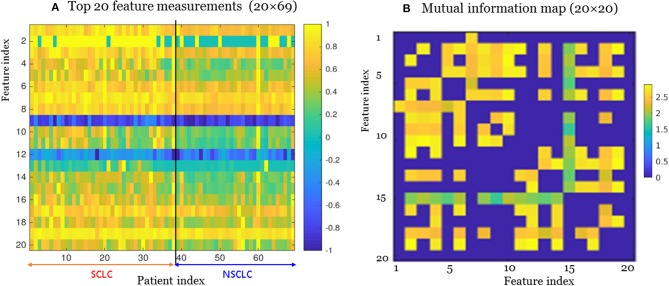
The top 20 features selected from the radiomic data set (total 1,731 features) for the small cell lung cancer (SCLC) / non-small-cell lung cancer (NSCLC) classification. **(A)** Measurements for top 20 features. Each feature (matrix row) consisted of 35 SCLC measurements (index 1:35) and 34 NSCLC measurements (index 36:69). Each feature vector was normalized by max=1. **(B)** Mutual information map for the top 20 features. A large mutual information value indicated a high redundancy between the features.

Using the mRMR method (30), we selected the most informative and non-redundant quantitative radiomic features. The correlation (Pearson) between two features assumed a value in the range [−1,1]. In this study, some feature correlations could approach 1 (e.g., among features extracted from different Ng values). For the high-correlation cases (e.g., corr>0.85), we removed one feature in the correlation pair and only kept the other feature (as done for feature selection). The feature selection and deselection procedure was implemented by a minimal-redundancy-maximal-relevance (mRMR) method. During feature selection, we removed one-feature in a high-correlation pair (e.g., corr>0.85), thereby removing the collinearity (corr~1). In lieu of a correlation map, we presented the mutual information map among the 20 features in Figure 3, which was used to present information redundancy, correlation, and dependence.

**Figure 4:**
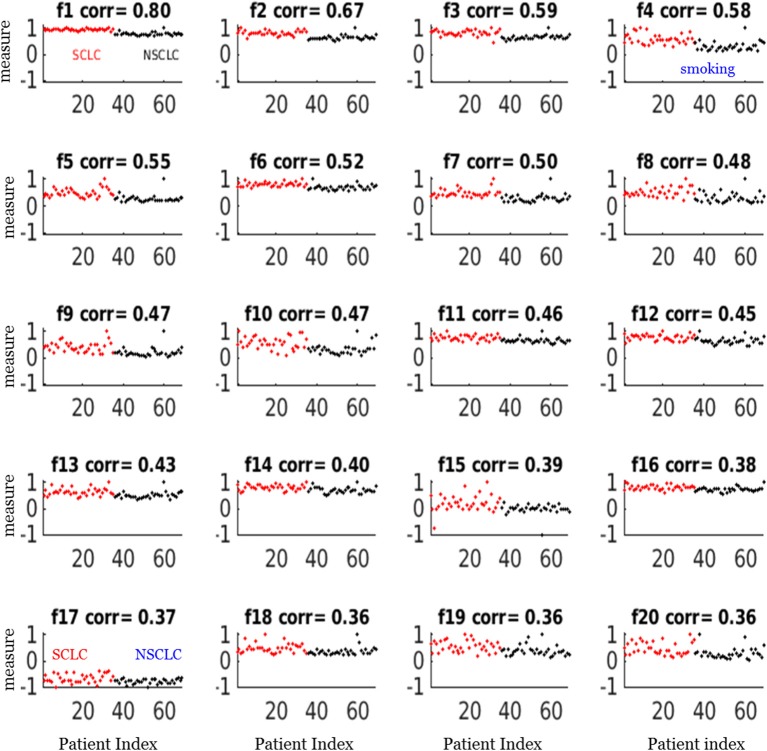
Scatter plots of the top 20 feature measurements from the dataset of 69 patients. All feature measures were normalized to a range [−1,1] (i.e., max = 1). The correlation (corr) value indicated the correlation between the feature vector and the target vector (SCLC = 1, NSCLC = 0). The notations for the selected features were presented in Table 2.

For our SCLC/NSCLC classifier, the top 20 features were selected from a total of 1731 features. In Figure 3A, we presented the selected 20 features representing 69 tumors. In Figure 3B, we presented the mutual information map. The selected features were also listed in Table 2. Notably, the clinical feature “smoking” was ranked fourth in the SCLC/NSCLC classification. Figure 4 contains a scatter graph for the top 20 features for inspection of the feature variability across the cohort. The features were sorted according to the correlation coefficient between the specific and the target vector (designated as the corr value).

**Table 2:** Top 20 features for SCLC/NSCLC classification in descending order of feature correlation with the target vector.

**(a) Top 20 features including clinical data**	**(b) Top 20 features excluding clinical data**
GLSZM.ZSN @ WT(HHH)	GLSZM.ZSN @ WT(HHH)
NGTDM.complex @ WT(LHL)	NGTDM.complex @ WT(LHL)
Global.range @ WT(LHH)	Global.range @ WT(LHH)
Smoking @ Clinic	GLSZM.SZLGE @ WT(LLH)
GLSZM.SZLGE @ WT(LLH)	GLSZM.LGZE @ WT(HHL)
GLSZM.LGZE @ WT(HHL)	GLSZM.ZSN @ WT(LHH)
GLSZM.ZSN @ WT(LHH)	GLSZM.SZLGE @ WT(HHH)
GLSZM.SZLGE @ WT(HHH)	GLSZM.SZLGE @ WT(HLH)
GLSZM.SZLGE @ WT(HLH)	Global.variance @ WT(HLH)
Global.variance @ WT(HLH)	Global.kurt @ WT(HLH)
Global.kurt @ WT(HLH)	GLSZM.GLN @ WT(LHH)
GLSZM.GLN @ WT(LHH)	GLSZM.ZSN @ rawNg=32
GLSZM.ZSN @ rawNg=32	GLSZM.ZP @ WT(HLH)
GLSZM.ZP @ WT(HLH)	GLSZM.ZSN @ WT(LLL)
GLSZM.ZSN @ WT(LLL)	Global.mean @ WT(HHL)
Global.mean @ WT(HHL)	NGTDM.complex @ WT(LHH)
NGTDM.complex @ WT(LHH)	Global.max @ WT(LLH)
Global.max @ WT(LLH)	NGTDM.contrast @ WT(HLH)
NGTDM.contrast @ WT(HLH)	GLSZM.ZSV @ WT(LHH)
GLSZM.ZSV @ WT(LHH)	GLSZM.ZP @ rawNg=16

*@, feature derived from image; GLCM, gray-level co-occurrence matrix; Global, whole image statistics (no spatial attributes); GLN, gray-level non-uniformity; GLRLM, gray-level run-length matrix; GLSZM, gray-level size zone matrix; LRLGE, long run low gray-level emphasis; raw, original CT image (no wavelet transform); SZLGE, small zone low gray-level emphasis; WT(xxx), wavelet-transform with 8 subbands (LLL, LLH, LHL, LHH, HLL, HLH, HHL, or HHH); ZP, zone percentage; ZSN, zone size non-uniformity; ZSV, zone size variance*.

### Classifier Performance

**Figure 5:**
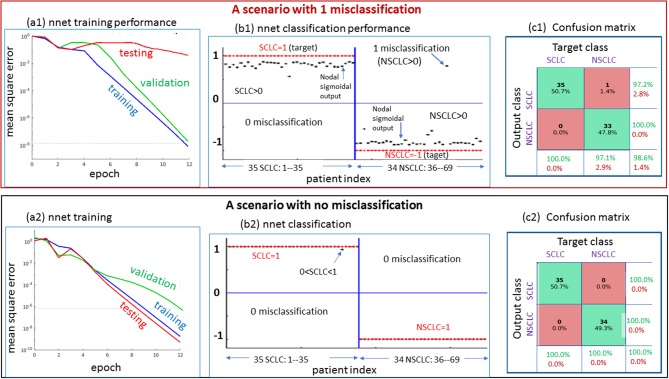
Two scenarios for demonstrating the *nnet* “training-validating-testing” performance. Upper: one case of 1 misclassification; lower: one case of no misclassification. The panels designated as a1 and a2 present the *nnet* training behaviors under random initial settings (w: weight and b: bias); The panels designated as b1 and b2 present the output node values (in value range [−1,1], in black dots) in reference to target setting (SCLC = 1, NSCLC = -1); and the panels designated as c1 and c2 present the confusion matrices. SCLC, small cell lung cancer; NSCLC, non-small cell lung cancer.

In Figure 5, we presented 2 scenarios demonstrating the *nnet* “training-validating-testing” performance. Specifically, in panels (a1,b1,c1), we showed a 1-misclassification case. As seen in panel (a1), the training and validation exhibited faster convergence than the testing. As seen in panel (b1), there was 1 misclassifiction for one NSCLC tumor (marked in arrow). As seen in panel (c1), the summary confusion matrix gave an accuracy of ~ 98%. In the output layer, the nodal sigmoid values (denoted by f, marked in black dots) approached the target class values (1 and−1), and the binary SCLC/NSCLC decision was made upon a thresholding (SCLC: f > 0, and NSCLC: f < 0, see illustration in Figure 2). With a similar layout in panels (a2,b2,c2), we presented a case of 0 misclassification with a 100% accuracy in the confusion matrix.

**Figure 6:**
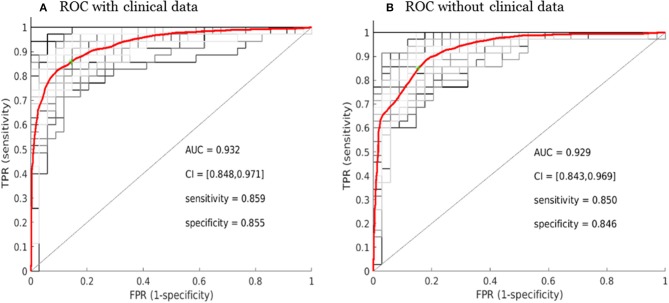
Receiver operating characteristic curve (ROC) performance for the SCLC/NSCLC neural network classifications with the clinical data **(A)** and without the clinical data **(B)**. The average ROC plot was the average over 30 ROC trials with random initializations for the classifier. *AUC*, area under the ROC curve; *FPR*, false positive rate; *TPR*, true positive rate; *CI*, confidence interval.

The overall performance of the SCLC/NSCLC classifier was presented in Figure 6A with clinical features and Figure 6B without clinical features. Our SCLC/NSCLC classification achieved an overall performance of AUC = ~0.93, sensitivity = 0.85, and specificity = 0.85. This classification performance also represented the prediction performance due to random partitioning of the cohort for constructing the classifier.

## Discussion

In this study, we present a CT radiomic model with a neural network classifier for differentiating SCLC from NSCLC adenocarcinoma with satisfying classification performance achieving an AUC of 0.93. We improved the model performance by including clinical data such as smoking history, which was relevant because smoking was a major risk factor for SCLC. Our top-ranking quantitative radiomic features for differentiating SCLC from NSCLC adenocarcinoma were mostly textural that was not perceptible to the human eye. Our study method presented the advantage of CT radiomics with computational algorithms being potentially outperforming the traditional human vision-based lung CT image assessment. Our study showed that CT radiomics could be potentially helpful to enhance our capability for tumor characterization and malignancy prediction.

Our study also showed that a combination of key radiomic features, rather than a single feature, could enhance classification performance of differentiating SCLC from NSCLC. For example, the best feature only attained a correlation coefficient of 0.80 in correlation with the target as shown in Figure 4 (f1), which was the measurement for linear vector discrimination, and the clinical feature “smoking” only attained a correlation coefficient of 0.6. However, by assembling the individual features into an ensemble including both radiomic and clinical features and then using the *nnet* nonlinear mapping, we built a robust SCLC/NSCLC classifier with reliable performance. It should be noted that the clinical feature “smoking” was ranked fourth in the SCLC/NSCLC classification and was included in the model building. However, the clinical feature “gender” was not sufficiently discriminative to be selected in the top 20 important features and therefore was not included for model building.

Our study results were generally in agreement with the literature. Linning et al. built four radiomic classification models using extracted radiomic features to evaluate the phenotypic differences between SCLC and NSCLC or NSCLC subtypes, and achieved an AUC of 0.82 (21). Linning et al. also indicated that the differences in the radiomic features may be correlated with subtle differences in tumor heterogeneity of the lung cancer histological subtypes. Our study had similar findings as theirs as most of our significant radiomic features were textural in nature reflecting tumor heterogeneity. In addition, these textural radiomic features were useful for differentiating primary lung cancer subtypes with subtle differences in tumor characteristics as in our cohort. Our study also showed that CT radiomics for SCLC/NSCLC differentiation was largely attributed to the power of computational CT image analysis with reproducible feature extraction, consistent texture assessment and the subsequent non-linear classifier via a multilayer neural network.

There were several limitations to this study. First, our exploratory pilot single-center study results of a small sample size without external validation may not be generalizable to other studies. In addition, one may have concern for reliable statistical inference since our classifier for radiomics-based lung cancer subtypes was developed from a small study cohort. Nevertheless, in dealing with the small sample size, we conducted a large number of repetitions of “training-validation-testing” procedure with random initial (weight, bias) settings and random sample set split for assessing the *nnet* performance. Second, our study used a tumor segmentation method that started with a semi-automatic approach utilizing a software to mark the regions of interest and then was supplemented with manual tracing of tumor boundaries. This method was time-consuming and required an imaging specialist throughout the segmentation process, which was susceptible to inter-observer and intra-observer variability (31). Nevertheless, the tumor segmentation step was performed by trained research staff and the tumors were carefully delineated slice-by-slice to minimize the segmentation errors that could be propagated to the subsequent radiomic modeling. For our future studies, we plan to test automated lung tumor segmentation, to incorporate a robust convolution neural network for predictive modeling and to develop a fully automated SCLC/NSCLC classifier.

Our study has also encountered several confounding factors inherent in a retrospective study including a heterogeneous study cohort, variability in imaging protocols and scanners, and non-standardized imaging reconstruction methods (32). This limitation may have caused subtle variations in the imaging features of the lung cancers and may have caused variabilities in tumor identification and segmentation. However, this was less an issue in our cohort of peripherally-located lung cancers because the clear demarcation and different tissue densities between the tumors and the surrounding lung parenchyma may have reduced ambiguity in the tumor segmentation step. Additionally, because our study was focused on radiomic feature extraction, we did not evaluate the semantic imaging features described by radiologists, such as location of the lung nodule, presence of emphysema, interstitial lung disease, pleural effusion, ground glass opacity, and nodule attenuation on the lung CT (33). These radiological features are usually obtained via human vision-based traditional imaging assessment which has been carried out in routine clinical practice. On the other hand, the radiomic analysis with computerized algorithms is mostly used in a research setting currently because it is not intuitive nor perceptible to human eyes. Nevertheless, combining these conventional radiological findings with radiomic features may improve the SCLC/NSCLC classifier performance, which we plan to do for our future research. Lastly, we did not perform radiomics-based classification on the lung cancer vs. the surrounding lung parenchyma or benign vs. malignant lung nodules. Future research is needed to assess the usefulness of radiomics for clinically relevant tasks such as classifying lung nodules vs. peri-nodular lung parenchyma (34, 35).

Despite the limitations, the promising results of our exploratory pilot study support moving forward with a large-scale multicenter study applying radiomics and artificial intelligence to precision medicine in the diagnosis and treatment of lung cancer. For our future study, we plan to perform radiogenomic analysis combining radiomics and genomic data to predict treatment response and survival in primary lung cancer. In addition, we will also aim to develop a more robust predictive modeling generalizable to other cancer types in addition to lung cancer in our future work.

In summary, our study showed that CT radiomic approach could potentially be used as a non-invasive imaging-based biomarker to differentiate primary lung cancer subtypes such as SCLC vs. NSCLC, thereby contributing to early diagnosis and treatment of lung cancer.

## Data Availability Statement

The raw data supporting the conclusions of this article will be made available by the authors, without undue reservation, to any qualified researcher.

## Ethics Statement

The studies involving human participants were reviewed and approved by Institutional Review Board for City of Hope National Medical Center. Written informed consent for participation was not required for this study in accordance with the national legislation and the institutional requirements.

## Author Contributions

BC and RS designed and conducted the study. BC, ZC, NY, IM, GW, and RS analyzed the brain MRI scan data and contributed to the manuscript writing process and BC prepared the first draft of the entire manuscript. ZC, NY, and BC performed tumor segmentation, radiomic feature extraction, feature selection, and classification. BC, ZC, NY, IM, JF, ED, GW, CW, RR, RC, MN, SB, AH, SS, and RS contributed to data interpretation. All authors approved the final manuscript.

## Conflict of Interest

AH reports personal fees from fMRI Consultants, LLC, outside the submitted work. The remaining authors declare that the research was conducted in the absence of any commercial or financial relationships that could be construed as a potential conflict of interest.
