# Supplementary material for: Differentiating Peripherally-Located Small Cell Lung Cancer From Non-small Cell Lung Cancer Using a CT Radiomic Approach
Source: Front Oncol. 2020 Apr 22;10:593. doi: 10.3389/fonc.2020.00593 (PMC7188953; doi:10.3389/fonc.2020.00593)

Supplementary Material

**Supplementary Figure 1 (Figure 1S).** Heat map of the 1731 features (including 1728 image features and 3 clinical features). The feature vectors (matrix rows) are normalized by max=1 as indicated by the scale bar. Each feature consists of 35 SCLC measurements and 34 NSCLC measurements, partitioned by the vertical line.


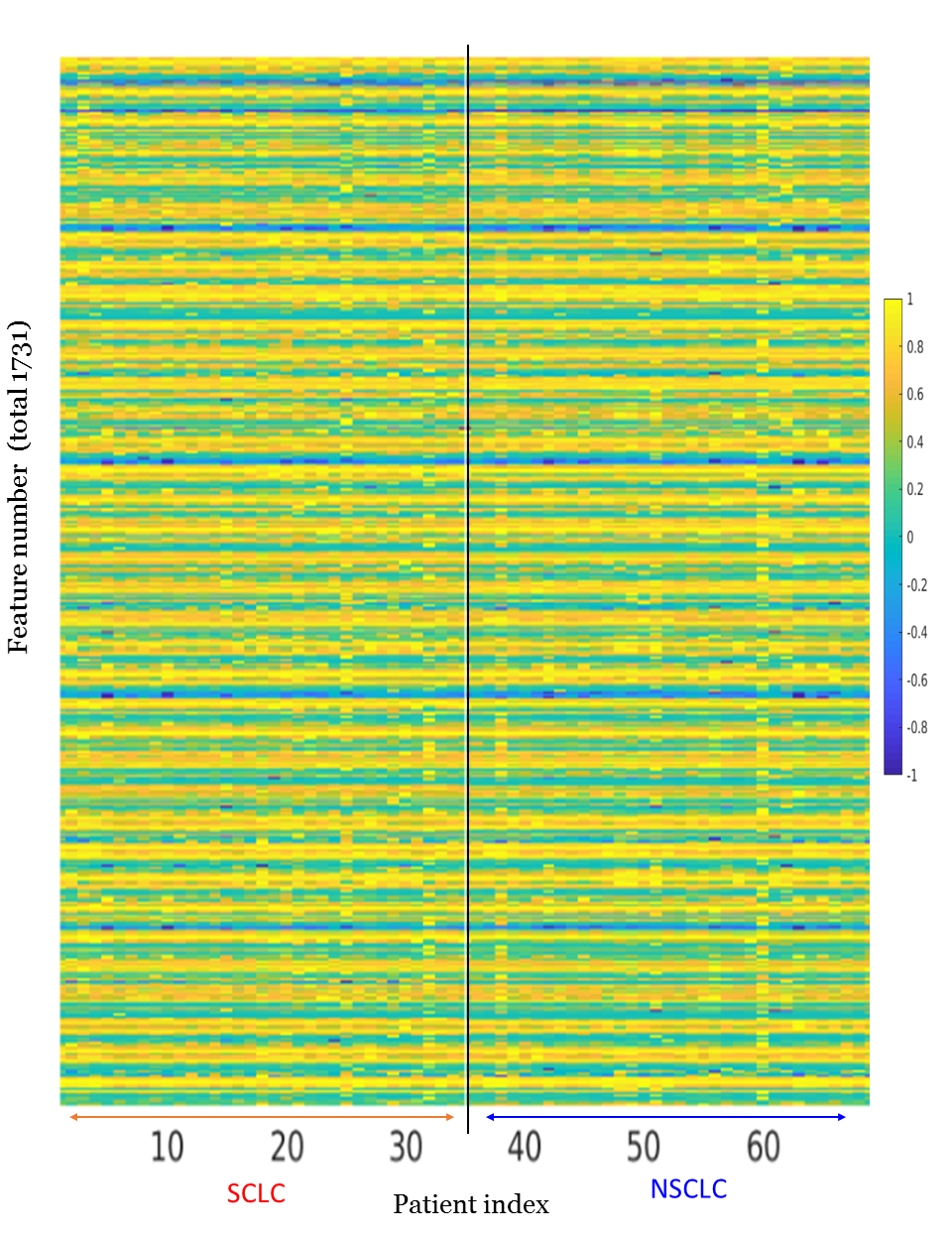


**Supplementary Figure 2 (Figure 2S).**  The mutual information map (in a 1731x1731 symmetrical matrix) of the radiomic features. The matrix is filled with large-value entries, indicating a prevailing high redundancy among the features.


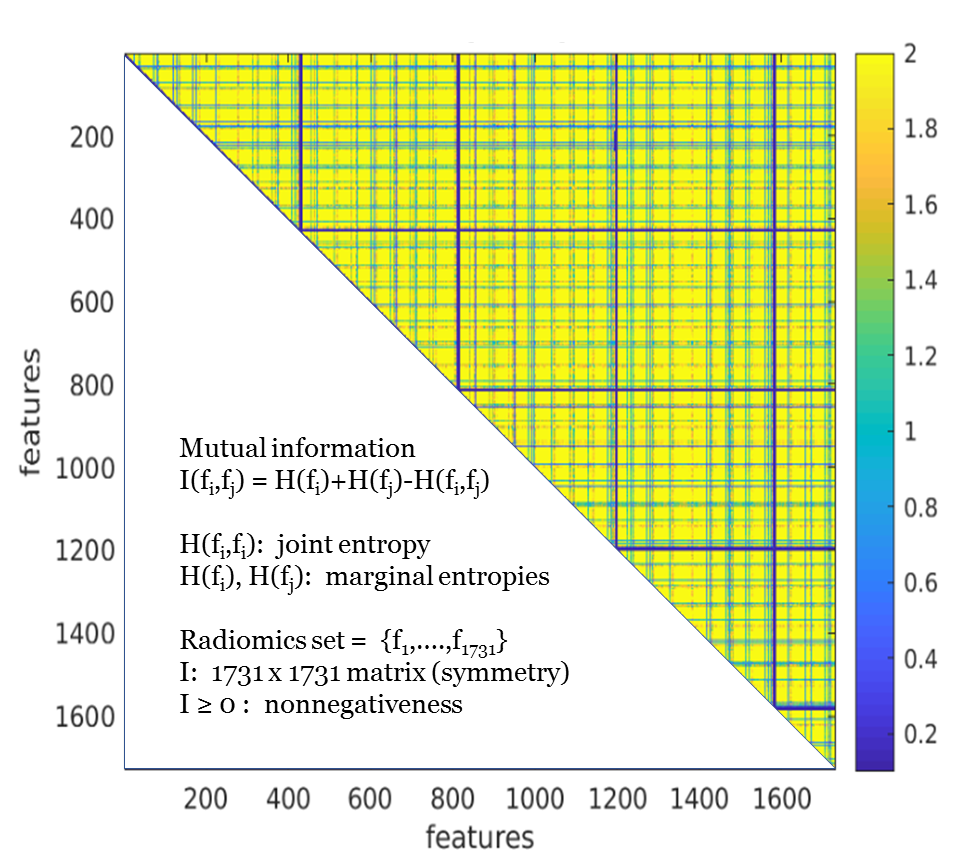

Supplement: Supplementary file 1 [file Table_1.DOCX]
